# Supplementary figures and images for: Comparison of the change in QuantiFERON-TB Gold Plus and QuantiFERON-TB Gold In-Tube results after preventive therapy for latent tuberculosis infection
Source: PLoS One. 2020 Jun 16;15(6):e0234700. doi: 10.1371/journal.pone.0234700 (PMC7297367; doi:10.1371/journal.pone.0234700)

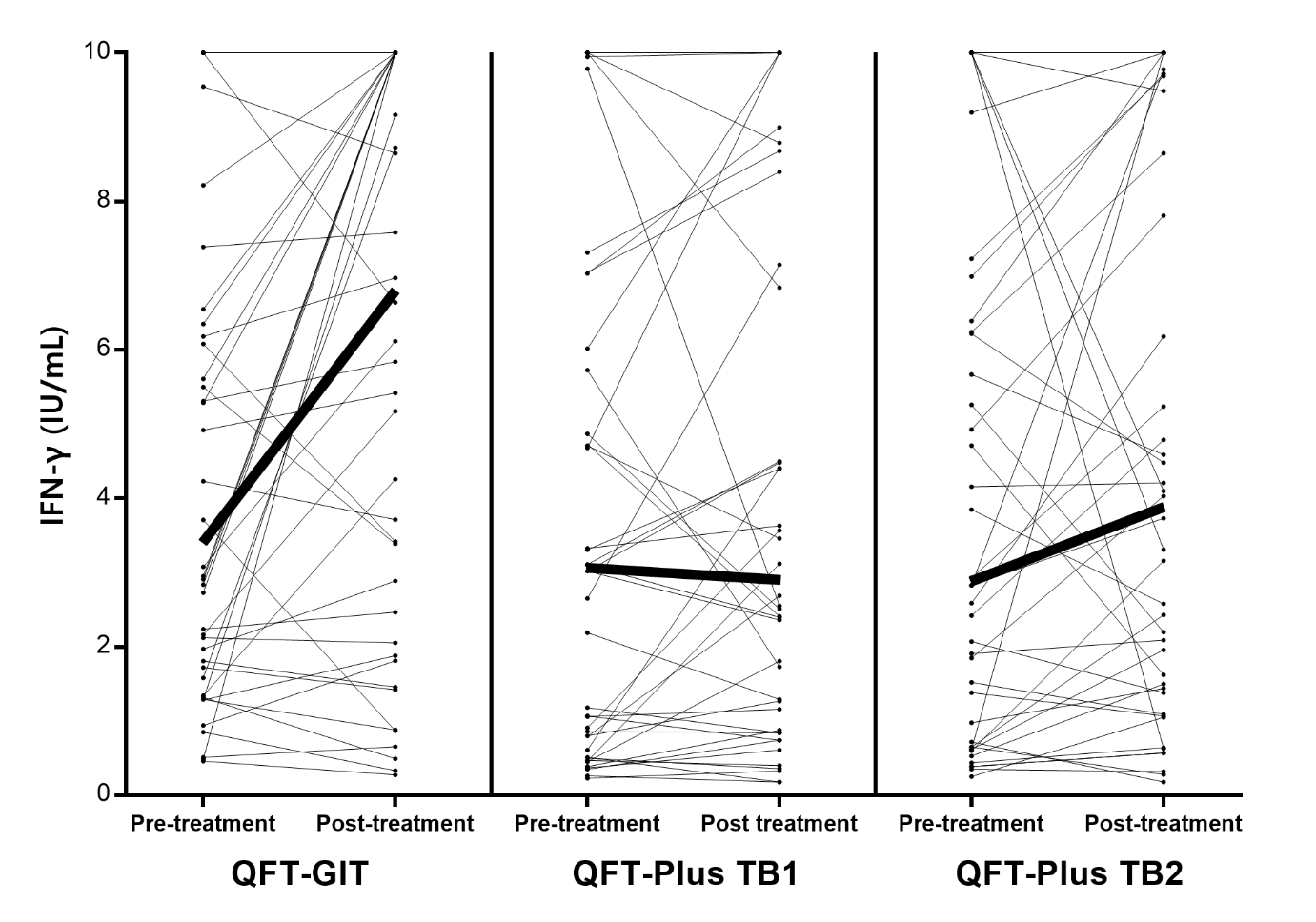

Supplement: S1 Fig — IFN-γ concentrations from each participant are shown (•). Median levels are indicated by heavy lines. IFN-γ, interferon-γ; QFT-GIT, QuantiFERON-TB Gold In-Tube; QFT-Plus, QuantiFERON-TB Gold Plus. (DOCX) [file pone.0234700.s001.docx]

**A**


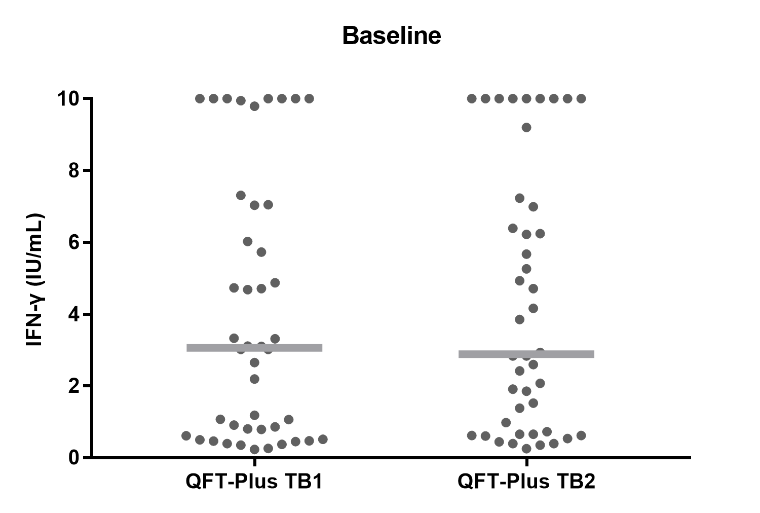

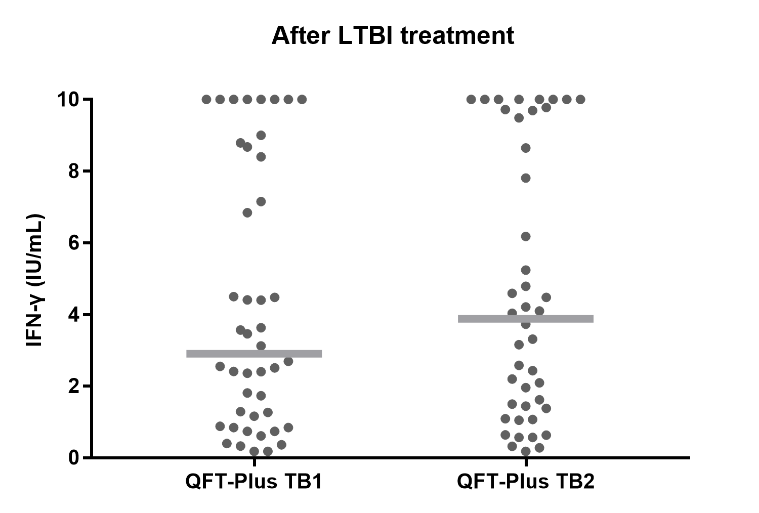


**B**


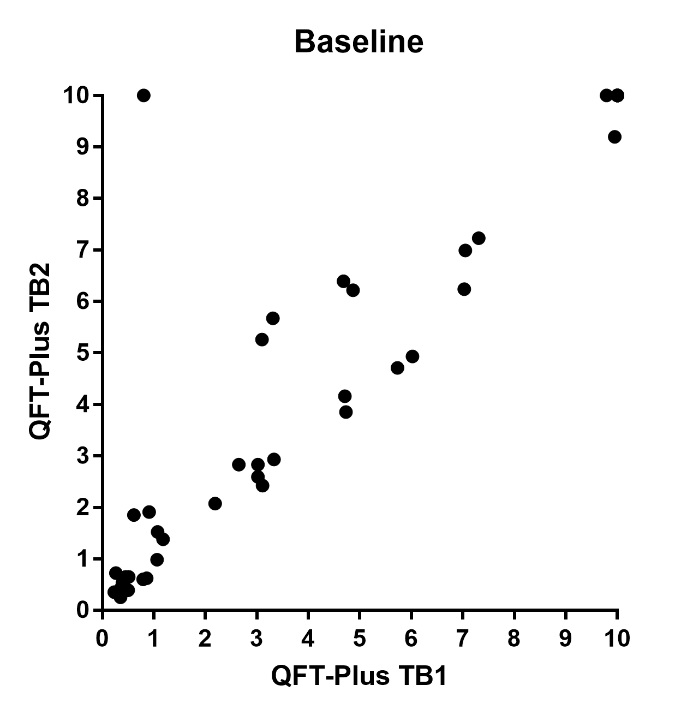

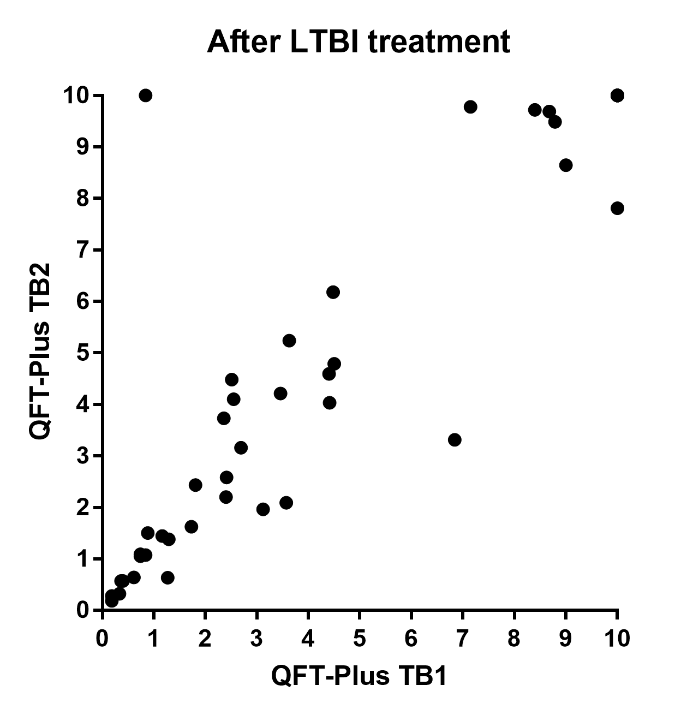

Supplement: S2 Fig — (A) IFN-γ response to QFT-Plus antigen TB1 and TB2 is similar both at baseline and after LTBI treatment. (B) Positive correlation between IFN-γ values in response to TB1 and TB2 stimulation both at baseline and after LTBI treatment (Baseline: r = 0.912, P < 0.001, after LTBI treatment: r = 0.892, P <0.001). IFN-γ, interferon-γ; QFT-Plus, QuantiFERON-TB Gold Plus; LTBI, latent tuberculosis infection. (DOCX) [file pone.0234700.s002.docx]

**A B**


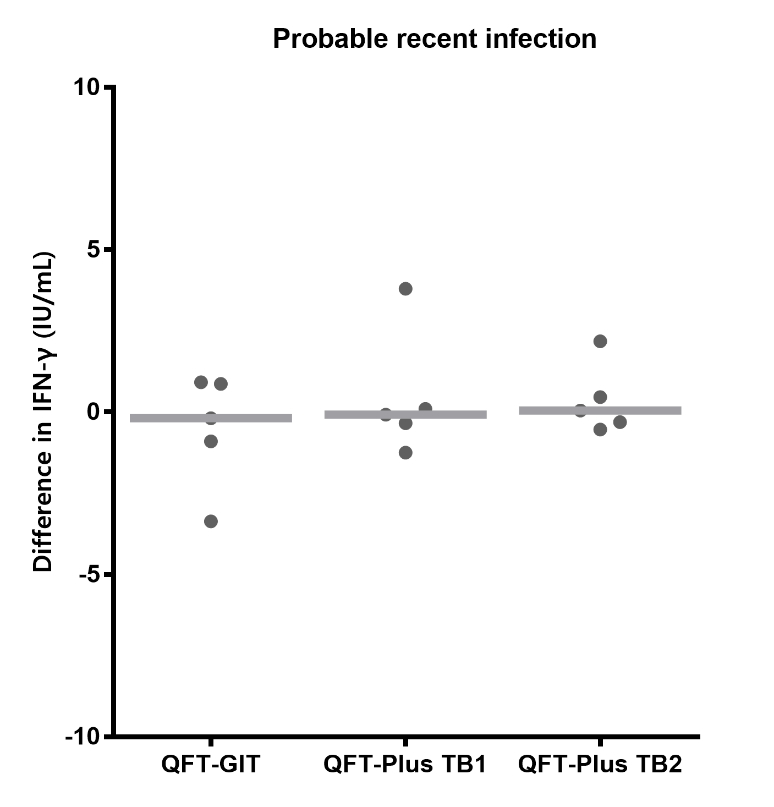

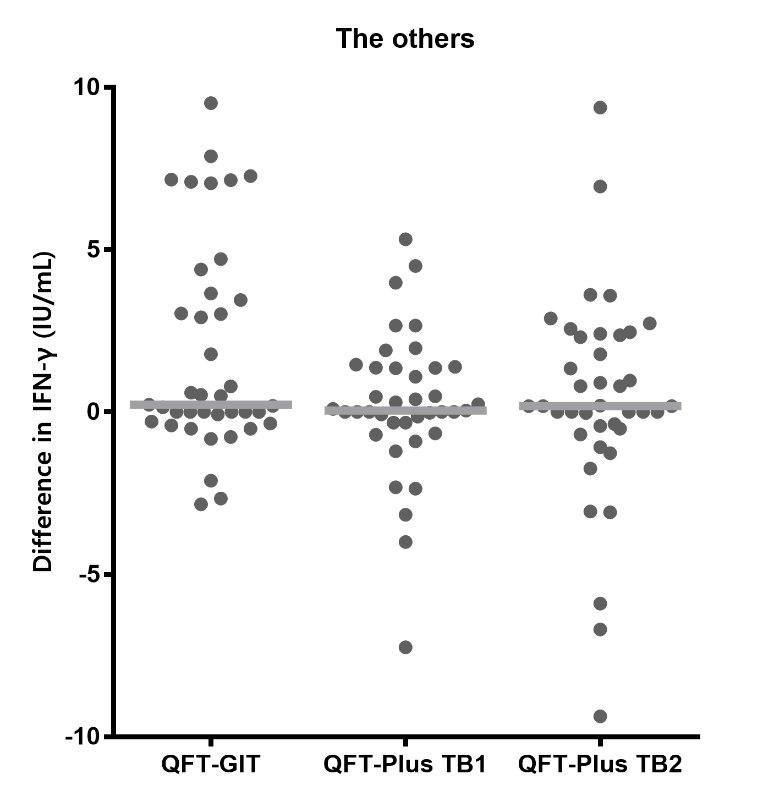

Supplement: S3 Fig — (A) The LTBI participants who were likely to have been infected recently; (B) the other participants. Difference in IFN-γ concentrations before and after preventive therapy for each participant are shown (●). Median levels are indicated by gray lines. IFN-γ, interferon-γ; QFT-GIT, QuantiFERON-TB Gold In-Tube; QFT-Plus, QuantiFERON-TB Gold Plus; LTBI, latent tuberculosis infection. (DOCX) [file pone.0234700.s003.docx]

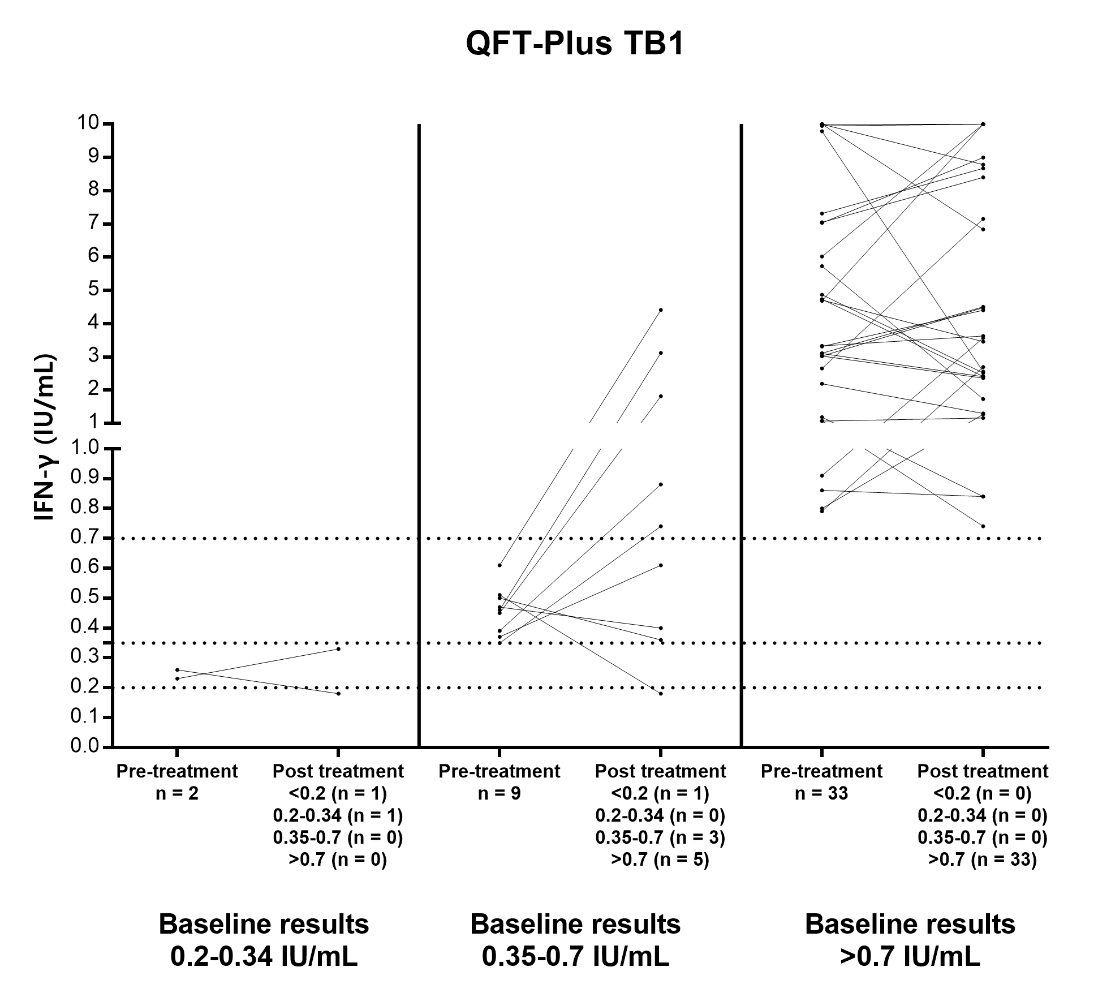


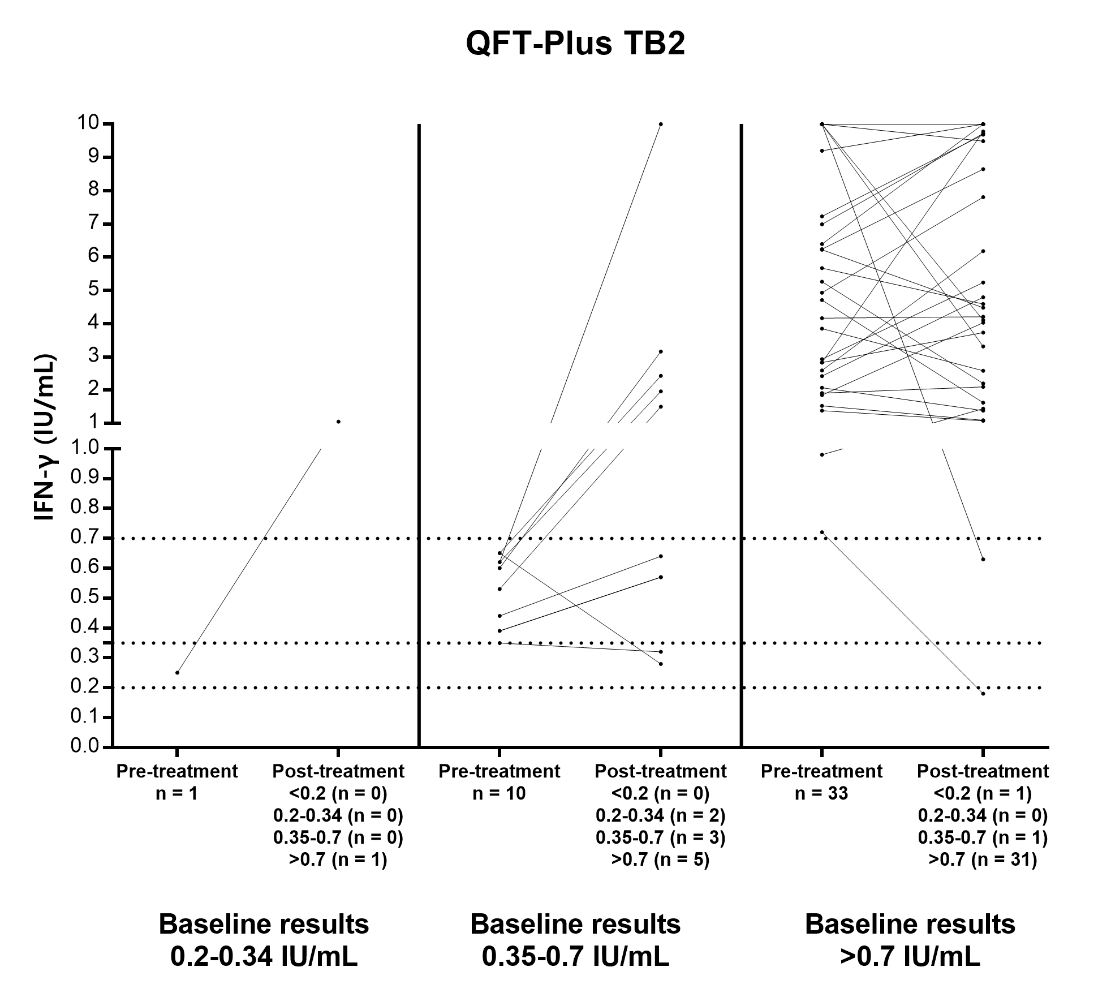

Supplement: S4 Fig — IFN-γ concentrations from each participant are shown (•). IFN-γ, interferon-γ; QFT-Plus, QuantiFERON-TB Gold Plus. (DOCX) [file pone.0234700.s004.docx]
