# Supplementary material for: Comparison of the change in QuantiFERON-TB Gold Plus and QuantiFERON-TB Gold In-Tube results after preventive therapy for latent tuberculosis infection
Source: PLoS One. 2020 Jun 16;15(6):e0234700. doi: 10.1371/journal.pone.0234700 (PMC7297367; doi:10.1371/journal.pone.0234700)
Supplement: S1 Table — (DOCX) [file pone.0234700.s005.docx]

|  | **QFT-GIT** | **QFT-Plus TB1** | **QFT-Plus TB2** |
| --- | --- | --- | --- |
| Before treatment | 3.395 | 3.060 | 2.880 |
| After treatment | 6.804 | 2.905 | 3.880 |
| *P*-value* | 0.008 | 0.247 | 0.167 |

Abbreviations: IFN-γ, interferon-γ; QFT-GIT, QuantiFERON-TB Gold In-Tube; QFT-Plus, QuantiFERON-TB Gold Plus. *Wilcoxon signed rank test.
