# Supplementary material for: Comparison of the change in QuantiFERON-TB Gold Plus and QuantiFERON-TB Gold In-Tube results after preventive therapy for latent tuberculosis infection
Source: PLoS One. 2020 Jun 16;15(6):e0234700. doi: 10.1371/journal.pone.0234700 (PMC7297367; doi:10.1371/journal.pone.0234700)
Supplement: Attachment — Submitted filename: letter.docx [file pone.0234700.s007.docx]

Dear Katalin Andrea Wilkinson,

Thank you very much for sending this manuscript to me. This paper is worth publishing; however, some major revision is recommended.

Special attention to response to TB1 or TB2 peptides should consider.

Recent review articles in this issue should consider.

[**Can Interferon-γ Release Assays Be Useful for Monitoring the Response to Anti-tuberculosis Treatment?: A Systematic Review and Meta-analysis**](https://link.springer.com/article/10.1007/s00005-020-00568-4)

[Archivum Immunologiae et Therapiae Experimentalis](https://link.springer.com/journal/5) volume 68, Article number: 4 (2020)

[**Comparison of the QuantiFERON-TB Gold Plus and QuantiFERON-TB Gold In-Tube interferon-γ release assays: A systematic review and meta-analysis**](https://www.sciencedirect.com/science/article/pii/S1896112618304218)

Advances in Medical Sciences. Volume 64, Issue 2, September 2019, Pages 437-443

How many had positive response to either TB1 or TB2 peptides?

The authors should stratify the QFT Plus results according to the ability of the subjects to respond to both TB1 and TB2 peptides (“TB1 and TB2”), only to TB1 (“only TB1”) or only to TB2 (“only TB2”).

The proportion of “only TB1” or “only TB2” responders change overtime or not?

Comparing the IFN-γ response to TB1 and TB2 peptides at the same time point is very important.

How was the median of TB1 and TB2 response at baseline and at the end of preventive therapy?

Did the IFN-γ production we stratified in LTBI subjects according to the time of exposure to Mtb?

In Petruccioli study, IFN-γ production decreased significantly in recent LTBI at the end of TB preventive therapy, in response to TB1 peptides. (see [**Effect of therapy on Quantiferon-Plus response in patients with active and latent tuberculosis infection**](https://www.nature.com/articles/s41598-018-33825-w.pdf?origin=ppub) Scientific reports | (2018) 8:15626)

it has been suggested the use of an uncertain zone for a better interpretation of the test resultsTo evaluate the distribution of IFN-γ values in the uncertain zone, authors should report their results stratifying the base-line results as follow: <0.2 IU/mL; ranging in 0.2–0.34 IU/mL; ranging in 0.35–0–7 IU/mL; >0.7 IU/Ml.

[**Effect of therapy on Quantiferon-Plus response in patients with active and latent tuberculosis infection**](https://www.nature.com/articles/s41598-018-33825-w.pdf?origin=ppub) Scientific reports | (2018) 8:15626
