# Supplementary material for: Comparison of the change in QuantiFERON-TB Gold Plus and QuantiFERON-TB Gold In-Tube results after preventive therapy for latent tuberculosis infection
Source: PLoS One. 2020 Jun 16;15(6):e0234700. doi: 10.1371/journal.pone.0234700 (PMC7297367; doi:10.1371/journal.pone.0234700)
Supplement: Attachment — Submitted filename: Response to reviewers.docx [file pone.0234700.s008.docx]

**To the Editor of** *PLOS ONE*

Dear Katalin Andrea Wilkinson:

We would like to thank you and the reviewers for the helpful comments pertaining to our manuscript entitled “Comparison of the change in QuantiFERON-TB Gold Plus and QuantiFERON-TB Gold In-Tube results after preventive therapy for latent tuberculosis infection” (manuscript ID: PONE-D-19-16329R1). We believe that the manuscript has been significantly improved after incorporation of the comments provided.

The manuscript has been revised in accordance with your suggestions. Our point-by-point responses to the reviewers’ comments are provided below.

We hope that the manuscript is now suitable for publication in *PLOS ONE.*

Sincerely,

Tae Sun Shim, MD, PhD

Address: Division of Pulmonary and Critical Care Medicine,

University of Ulsan College of Medicine, Asan Medical Center,

88 Olympic-ro 43-gil, Songpa-gu, Seoul 05505, South Korea

Phone: 82-2-3010-3892; Fax: 82-2-3010-6968

E-mail: [shimts@amc.seoul.kr](mailto:shimts@amc.seoul.kr)

**Responses to Reviewer 1:**

There remain just a few minor revisions but otherwise the manuscript is acceptable for publication:

1. Statistics for figure 2- While the authors redid their statistics to find that there were differences in the results of baseline IFN-measurement between QFT-TB, TB1-QFT-plus and TB QFT plus. This would not be unusual given that the antigens used in each assay were different. While this fact may be fine for the results section of the manuscript, it may not be that critical for the abstract. Because the “objective was to assess whether QFT-Plus can serve as a biomarker of LTBI treatment response”, at a minimum the abstract should state either the number (and percentage) of positive results of QFT-PLUS and QFT-TB in the pre LTBI and post LTBI treatment groups to address the primary objective.

🡪 Thank you for your valuable comments. We have deleted the sentence about the baseline IFN-γ levels and have added the following text, as well as Table 2, concerning the number (and percentage) of positive results of the QFT-Plus and QFT-GIT assays after LTBI treatment in the Abstract and the Results sections of the revised manuscript.

“**Results:** The mean age of the participants was 47.6 years. **The QFT-GIT and QFT-Plus assays revealed positive results in 42/44 (95.5%) and 41/44 (93.2%) participants after LTBI treatment, showing overall agreement of 93.2%, with a Cohen’s kappa value of 0.37 (fair agreement).** The differences between pre- and post-LTBI treatment interferon-γ levels were measured using the QFT-GIT and QFT-Plus assays. No significant differences were noted among the 3 values: the median difference in interferon-γ value with QFT-GIT, QFT-Plus TB1, and QFT-Plus TB2 was 0.211 IU/mL (IQR, −0.337–3.347), 0.025 IU/mL (IQR, −0.338–1.368), and 0.180 IU/mL (IQR, −0.490–2.278), respectively (*P* = 0.401).”

(Page 2, lines 33–35 in the Abstract of the revised manuscript)

“**Table 2. The results of QFT assays at baseline and after the completion of preventive therapy.**

|  | **At baseline** | **After LTBI treatment** |
| --- | --- | --- |
|  | **Positive N (%)** | **Positive N (%)** |
| **QFT-GIT** | 44 (100) | 42 (95.5) |
| **QFT-Plus TB1 or TB2** | 44 (100) | 41 (93.2) |
| **QFT-Plus TB1 and TB2** | 41 (93.2) | 41 (93.2) |
| **QFT-Plus only TB1** | 1 (2.3) | 0 (0) |
| **QFT-Plus only TB2** | 2 (4.5) | 0 (0) |

**Abbreviations: QFT, QuantiFERON; LTBI, latent tuberculosis infection; QFT-GIT, QuantiFERON-TB Gold In-Tube; QFT-Plus, QuantiFERON-TB Gold Plus.**”

(Page 8–9, lines 168–172 in the Results section of the revised manuscript)

“**After LTBI treatment, the QFT-GIT and QFT-Plus assays revealed positive results in 42/44 (95.5%) and 41/44 (93.2%) participants. Among the 44 participants with positive QFT-Plus assay results at baseline, 41/44 (93.2%) responded to both TB1 and TB2 at baseline, and the proportion did not change after treatment completion. In the remaining 3 participants, the QFT-Plus assay reverted to negative after LTBI treatment. The detailed results of the QFT-Plus according to the participants who responded to TB1 and/or TB2 peptides are shown in Table 2.**”

(Page 9–10, lines 191–196 in the Results section of the revised manuscript)

2. In the revised manuscript line 259-261, the authors state “the decline of the TB1 and TB2 responses on follow tests in the subjects with positive QFT assays in the present study” is not true. These were not statically significant and therefore this statement should be modified or removed from the revised manuscript.

🡪 We have modified the text in the Discussion section of the revised manuscript as follows:

“**Although we generally did not observe a statistically significant trend in terms of changes in IFN-γ level before and after LTBI treatment, some participants had a reduction in the TB1 and/or TB2 response on follow-up tests. This decline could be attributed to the natural course of IFN-γ response rather than to the effects of LTBI treatment, considering the findings of a previous study showing that approximately half of the contacts of patients with TB with initial positive ELISPOT assay had shown a negative result on repeat ELISPOT assay at 3 months, in the absence of treatment [29].**”

(Page 14, lines 292–298 in the Discussion section of the revised manuscript)

**Responses to Reviewer 3:**

This paper is worth publishing; however, some major revision is recommended.

Special attention to response to TB1 or TB2 peptides should consider.

Recent review articles in this issue should consider.

1. How many had positive response to either TB1 or TB2 peptides?

🡪 Thank you for your valuable comments.

1) First of all, before we revise our present study, we want to highlight some of the differences between the present study and the study, “Effect of therapy on Quantiferon-Plus response in patients with active and latent tuberculosis infection,” on which you commented (Petruccioli E, Chiacchio T, Vanini V, et al. Effect of therapy on Quantiferon-Plus response in patients with active and latent tuberculosis infection. Sci Rep. 2018; 8: 15626.).

In the Petruccioli study, they have analyzed whether the overall median IFN-γ levels before and after treatment measured using only QFT-Plus were significantly different, using the Wilcoxon signed rank test.

However, the present study measured the median value of the differences between pre- and post-treatment IFN-γ to evaluate the change in each individual. Moreover, we compared the difference in the IFN-γ value measured using the QFT-Plus assay (TB1 or TB2) with the QFT-GIT result using the Friedman test.

2)

The results of the QFT-Plus TB1 and QFT-Plus TB2

| **TB2 result** | **TB1 result** | |
| --- | --- | --- |
|  | Positive | Negative |
| Positive | 41 | 2 |
| Negative | 1 | 0 |

As shown in the Table above, among the 44 participants enrolled in our study, most (41/44, 93.2%) were positive with both QFT-Plus TB1 and TB2, with the exception of 3 participants, 1 of who was positive with QFT-Plus TB1 only, whereas the other 2 were positive with QFT-Plus TB2 only.

We have thus added the following text to the Results section of the revised manuscript:

“**Among the 44 participants, most (41/44, 93.2%) were positive with both QFT-Plus TB1 and TB2, with the exception of 3 participants, 1 of who was positive with QFT-Plus TB1 only, whereas the other 2 were positive with QFT-Plus TB2 only (Table 2)**.”

(Page 7, lines 154–157 in the Results section of the revised manuscript)

2. The authors should stratify the QFT Plus results according to the ability of the subjects to respond to both TB1 and TB2 peptides (“TB1 and TB2”), only to TB1 (“only TB1”) or only to TB2 (“only TB2”). The proportion of “only TB1” or “only TB2” responders change overtime or not?

🡪 We have stratified the study participants according to the results of the QFT-Plus assay with 2 different TB antigen tubes (TB1 and TB2).

Among the 44 participants with positive QFT-Plus assay results, 41/44 (93.2%) responded to both TB1 and TB2 at baseline, and the proportion did not change at the end of therapy. The proportion of only-TB1 and only-TB2 responders changed to 0% over time. In other words, 3 participants had QFT-Plus reversions after LTBI treatment. The participant (1/41, 2.4%) who had responded to both TB1 and TB2 and the other 2 (2/2, 100%) participants who had responded to only TB2 at baseline showed QFT-Plus reversion after LTBI treatment. The participant who had responded to only TB1 at baseline responded to both TB1 and TB2 at the end of therapy, and reversion was not observed.

We have added Table 2 and the following text to the Results section of the revised manuscript.

“**Table 2. The results of QFT assays at baseline and after the completion of preventive therapy.**

|  | **At baseline** | **After LTBI treatment** |
| --- | --- | --- |
|  | **Positive N (%)** | **Positive N (%)** |
| **QFT-GIT** | 44 (100) | 42 (95.5) |
| **QFT-Plus TB1 or TB2** | 44 (100) | 41 (93.2) |
| **QFT-Plus TB1 and TB2** | 41 (93.2) | 41 (93.2) |
| **QFT-Plus only TB1** | 1 (2.3) | 0 (0) |
| **QFT-Plus only TB2** | 2 (4.5) | 0 (0) |

**Abbreviations: QFT, QuantiFERON; LTBI, latent tuberculosis infection; QFT-GIT, QuantiFERON-TB Gold In-Tube; QFT-Plus, QuantiFERON-TB Gold Plus.**”

(Page 8–9, lines 168–172 in the Results section of the revised manuscript)

“**Among the 44 participants with positive QFT-Plus assay results at baseline, 41/44 (93.2%) responded to both TB1 and TB2 at baseline, and the proportion did not change after treatment completion. In the remaining 3 participants, the QFT-Plus assay reverted to negative after LTBI treatment. The detailed results of the QFT-Plus according to the participants who responded to TB1 and/or TB2 peptides are shown in Table 2.**”

(Page 9–10, lines 192–196 in the Results section of the revised manuscript)

3. Comparing the IFN-γ response to TB1 and TB2 peptides at the same time point is very important.

🡪 We have analyzed IFN-γ response to TB1 and TB2 stimulation at baseline and after LTBI treatment.

As shown the Figure above, comparing the IFN-γ response to TB1 and TB2 peptides at the same time point, we observed similar levels of IFN-γ.

In addition, we found a positive correlation between the IFN-γ values in response to TB1 and TB2 stimulation both at baseline and after LTBI treatment (Baseline: r = 0.912, *P* < 0.001; after LTBI treatment: r = 0.892, *P* <0.001), as described in the Petruccioli study.

We have added the following text and the Figure above to the Results section of the revised manuscript:

“**In addition, when we compared the IFN-γ response to TB1 and TB2 peptides at the same time point, we observed similar levels of IFN-γ and a positive correlation between IFN-γ values in response to TB1 and TB2 stimulation both at baseline and after LTBI treatment. (S2 Fig).**”

(Pages 11, lines 225–228 in the Results section of the revised manuscript)

4. How was the median of TB1 and TB2 response a baseline and at the end of preventive therapy?

🡪 In addition to the median difference in the IFN-γ levels before and after LTBI treatment, we also re-analyzed whether the overall median IFN-γ levels before and after treatment were significantly different. We have represented the IFN-γ levels as median values because the data were not normally distributed.

The median IFN-γ levels before and after preventive therapy:

|  | **QFT-GIT** | **QFT-Plus TB1** | **QFT-Plus TB2** |
| --- | --- | --- | --- |
| Before treatment | 3.395 | 3.060 | 2.880 |
| After treatment | 6.804 | 2.905 | 3.880 |
| *P*-value | 0.008 | 0.247 | 0.167 |

As shown in the Figure and Table above, median IFN-γ levels measured using the QFT-GIT and QFT-Plus TB2 assays increased between baseline and the end of LTBI treatment (Wilcoxon signed rank test *P* = 0.008, 0.167, respectively). Although they were not statistically significant, the median IFN-γ levels of the QFT-Plus TB1 assay decreased after LTBI treatment (Wilcoxon signed rank test *P* = 0.247).

We have added the following sentence, as well as the Figure and Table above, to the Results section of the revised manuscript.

“**We also evaluated the overall median IFN-γ levels before and after treatment, the results of which are shown in the supporting information (S1 Fig and S1 Table)**.”

(Page 11, lines 223–225 in the Results section of the revised manuscript)

5. Did the IFN-γ production we stratified in LTBI subjects according to the time of exposure to Mtb? In Petruccioli study, IFN-γ production decreased significantly in recent LTBI at the end of TB preventive therapy, in response to TB1 peptides. (see Effect of therapy on Quantiferon-Plus response in patients with active and latent tuberculosis infection Scientific reports | (2018) 8:15626)

🡪 In the Petruccioli study, the subjects with LTBI were divided into those with remote infection (at least 3 years) and those with recent infection (no more than 3 months). However, as described at line 281–284 in the Discussion section of the revised manuscript, the majority of the participants in our study were healthcare and day care center workers or candidates for TNF inhibitor therapy, and we could not evaluate the time of exposure *to M. tuberculosis*.

Therefore, we divided the study participants into the those who were likely to have been infected recently (4 participants in close contact with people with tuberculosis and 1 recently converted) and the other participants, then observed IFN-γ production.

1) Among the 5 participants who were likely to have been infected recently, 4/5 (80.0%) showed a positive response in QFT-GIT and QFT-Plus (TB1 or TB2) after the completion of therapy, whereas in the other group, 35/36 (97.2%) and 34/36 (94.4%) responded after the treatment in QFT-GIT, and QFT-Plus (TB1 or TB2), respectively.

2)

The median IFN-γ levels before and after preventive therapy in the LTBI participants with probable recent infection and the other participants:

| **Probable recent infection** | **QFT-GIT** | **QFT-Plus TB1** | **QFT-Plus TB2** |
| --- | --- | --- | --- |
| Before treatment | 1.971 | 1.060 | 1.380 |
| After treatment | 2.888 | 1.160 | 1.440 |
| *P*-value | 0.686 | 0.893 | 0.686 |

| **The other participants** | **QFT-GIT** | **QFT-Plus TB1** | **QFT-Plus TB2** |
| --- | --- | --- | --- |
| Before treatment | 3.710 | 3.110 | 3.850 |
| After treatment | 7.584 | 3.120 | 4.100 |
| *P*-value | 0.004 | 0.248 | 0.209 |

Analyzing the quantitative results, the median IFN-γ levels measured using the QFT-GIT assay, QFT-Plus TB1, and QFT-Plus TB2 increased between baseline and the end of LTBI treatment in the LTBI participants with probable recent infection (Wilcoxon signed rank test *P* = 0.686, 0.893, 0.686, respectively).

In the other group, the median IFN-γ levels measured using the QFT-GIT assay, QFT-Plus TB1, and QFT-Plus TB2 also increased between baseline and the end of LTBI treatment (Wilcoxon signed rank test *P* = 0.004, 0.248, 0.209, respectively) (See the Figures and Tables above).

3)

As shown in the Figures above, in the LTBI participants who were likely to have been infected recently, the median difference of the IFN-γ value of QFT-GIT was −0.187 IU/mL (IQR, −2.131–0.895), the median difference of TB1 QFT-Plus antigen IFN-γ level was −0.080 IU/mL (IQR, −0.795–1.950), and the median difference of TB2 QFT-Plus antigen IFN-γ level was 0.050 IU/mL (IQR, −0.425–1.320) (*P* = 0.819).

In the other group, the median difference of the IFN-γ value of QFT-GIT was 0.228 IU/mL (IQR, −0.296–3.650), the median difference of TB1 QFT-Plus antigen IFN-γ level was 0.050 IU/mL (IQR, −0.330–1.370), and the median difference of TB2 QFT-Plus antigen IFN-γ level was 0.180 IU/mL (IQR, −0.510–2.370) *(P* = 0.454).

We have added the following sentence and the Figures above to the Discussion section of the revised manuscript:

“**Although the findings were generally the same when we stratified our participants according to the possibility of recent infection (S3 Fig), it was likely that the number of recently infected participants was too small to reveal statistical significance.**”

(Page 14, line 288–291 in the Discussion section of the revised manuscript)

6. It has been suggested the use of an uncertain zone for a better interpretation of the test results. To evaluate the distribution of IFN-γ values in the uncertain zone, authors should report their results stratifying the base-line results as follow: <0.2 IU/mL; ranging in 0.2–0.34 IU/mL; ranging in 0.35–0–7 IU/mL; >0.7 IU/Ml. (see Effect of therapy on Quantiferon-Plus response in patients with active and latent tuberculosis infection Scientific reports | (2018) 8:15626)

🡪 Considering the inherent variability of the IGRA test, we performed our analysis again after stratifying our participants according to the baseline IFN-γ value of QFT-Plus (either TB1 or TB2), as previously described in the Petruccioli study: 0.2–0.34 IU/mL; 0.35–0.7 IU/mL; and >0.7 IU/mL. We found that 75% (33/44) of the participants had a baseline IFN-γ value >0.7 IU/mL in the QFT-Plus TB1 and TB2 assay, respectively.

As shown in the Figure above, among 3 participants who had QFT-Plus reversions after LTBI treatment, 2 were in the uncertainty zone, which had been defined as baseline IFN-γ value 0.2–0.7 IU/mL in a previous study (Nemes E, Rozot V, Geldenhuys H, et al. Optimization and interpretation of serial QuantiFERON testing to measure acquisition of *Mycobacterium tuberculosis* infection. Am J Respir Crit Care Med. 2017; 196: 638–648.). The majority of the LTBI participants with IFN-γ values at baseline >0.7 IU/mL also had a response >0.7 IU/mL after the completion of preventive therapy.

We have added the following text, as well as the Figure above, to the Discussion section of the revised manuscript:

“**Considering this inherent IGRA test variability, we performed our analysis again after stratifying our participants according to the baseline IFN-γ value of QFT-Plus (either TB1 or TB2) as previously described [25]: 0.2–0.34 IU/mL; 0.35–0.7 IU/mL; and >0.7 IU/mL. The overall results are shown in the supporting information (S4 Fig). Reversions tended to be more in the uncertainty zone, which had been defined as baseline IFN-γ value 0.2–0.7 IU/mL in previous studies [25,33]. This result suggests that some of the reversions could be attributable to inherent variability.”**

(Page 14–15, lines 308–314 in the Discussion section of the revised manuscript)
